# Surviving Postpartum Group A Streptococcus Sepsis Complicated by Multiorgan System Failure: A Complex Case Presentation

**DOI:** 10.7759/cureus.56167

**Published:** 2024-03-14

**Authors:** Rim Saab, Sarah Assali, Mary Angelides, Jay Idler

**Affiliations:** 1 Obstetrics and Gynecology, Drexel University College of Medicine, Philadelphia, USA; 2 General Surgery, Allegheny Health Network, Pittsburgh, USA; 3 Obstetrics and Gynecology, Allegheny Health Network, Pittsburgh, USA

**Keywords:** clinical management, multidisciplinary care, cardiomyopathy, toxic shock syndrome, peripartum sepsis, group a streptococcus

## Abstract

Postpartum group A streptococcal (GAS) sepsis is a rare obstetric complication with severe clinical implications and high morbidity and mortality, presenting diagnostic and management challenges. This report analyzes a complex case of postpartum GAS sepsis, highlighting the importance of understanding the pathophysiology and clinical trajectories of this often fatal pathogen. A comprehensive analysis was conducted on a patient with postpartum GAS sepsis. Literature review and case comparisons informed the study's context. Medical history, clinical presentation, diagnostic procedures, interventions, and outcomes were reviewed and documented. The patient presented on postpartum day 5 with abdominal pain and vaginal bleeding. Her condition rapidly deteriorated, requiring aggressive interventions and systemic support. Blood cultures confirmed GAS bacteremia. She developed toxic shock syndrome, cardiomyopathy with acute cardiac failure, and seizures secondary to subdural empyema. Multidisciplinary care facilitated eventual clinical recovery. Obstacles in achieving treatment balance were evident, underscoring the systemic nature of GAS infection and the significance of interdisciplinary collaboration. This case underscores the complex pathophysiology of postpartum GAS sepsis and the importance of prompt treatment initiation, aggressive intervention, and a multidisciplinary approach to management. The study contributes to the understanding of disease progression and clinical management in severe peripartum infections, reaffirming the need for further research to improve outcomes.

## Introduction

Peripartum group A streptococcus (GAS) sepsis is a rare yet highly morbid cause of puerperal sepsis that poses significant challenges in diagnosis and management [1-3]. Despite clinical advances, systemic consequences of invasive GAS infections have a propensity to escalate rapidly, affecting multiple organ systems and leading to severe morbidity and mortality [4-6]. This is especially true in the peripartum population, with postpartum women carrying upwards of 20 times the risk of their non-pregnant counterparts [7].

GAS has been implicated in a broad spectrum of infectious processes, ranging from minor pharyngitis to life-threatening invasive processes, such as necrotizing fasciitis and toxic shock syndrome (TSS) [8]. Targeted preventative efforts and management strategies have resulted in decreased incidence rates, including improved sanitation, risk factor mitigation, early antibiotic initiation, and heightened clinical vigilance [1,3,9]. Despite its low incidence and in light of many recently reported cases revealing an unexplained resurgence, treatment of peripartum GAS sepsis remains arduous. Many studies have shed light on the intricate interplay of microbial and host factors contributing to the pathogenesis of postpartum GAS infections. Disrupted mucocutaneous barriers, variation in vaginal pH, and alterations in immune responses have been identified as key contributors to the susceptibility of peripartum females [2,3,6,7,10,11]. Certain bacterial virulence factors have also been identified; specific M protein types have notably been associated with increased disease severity [1-4]. The increased vulnerability of peripartum women to bacterial infections is further compounded by their often atypical manifestations of sepsis [12]. These issues highlight the importance of early recognition and prompt interventions targeting invasive GAS infections in this population.

Peripartum GAS infections have a unique ability to escalate to severe sepsis, TSS, and multiorgan system failure [1,2,6,10]. GAS infections can rarely lead to subdural empyema (SDE) [13]. In rare cases of SDE, subdural collections are identified during later imaging studies, further demonstrating the importance of clinical vigilance [13]. Fortunately, with early and aggressive surgical intervention, these patients can experience full neurologic recovery without persistent deficits [13].

Despite its infrequency, familiarity with manifestations of GAS infections is necessary for prompt diagnosis and employment of treatment strategies. This involves comprehensive measures such as blood and urine cultures, evaluation of lactic acid, endometrial sampling for culture, early administration of broad-spectrum antibiotics, and low threshold for surgical intervention [5]. Once GAS infection has been confirmed, targeted antibiotic therapy with high-dose penicillin and clindamycin is recommended [3,5].

Considering the complexity of peripartum GAS infections, it is crucial to better understand the host-pathogen interactions, immune responses, and clinical courses that define this condition. This case report reviews a severe case of postpartum GAS sepsis in a 42-year-old woman, complicated by TSS, Takotsubo cardiomyopathy, cardiac failure, and the development of SDE from hematogenous spread. This contributes to the current literature by presenting a multifaceted case of postpartum GAS sepsis, its rare sequelae of SDE, and the wide range of interdisciplinary collaboration central to her recovery. As we strive to improve maternal outcomes, it is essential to have a comprehensive approach to diagnosing, treating, and preventing postpartum GAS infections.

## Case presentation

A 42-year-old female with a remote history of seizure disorder and congenital deafness presented to an outside facility five days postpartum from a vaginal delivery with abdominal pain and vaginal bleeding. Upon arrival, she was noted to have tachycardia and hypotension despite intravenous fluid resuscitation. Initial laboratory results showed leukopenia (white blood cell count of 1,500/µL), anemia (hemoglobin level of 10.3 g/dL), and lactic acidosis (lactate level of 3.4 mmol/L). Transthoracic echocardiography (TTE) demonstrated severe left ventricular hypokinesis with a left ventricular ejection fraction (EF) of 15%. Abdominopelvic computed tomography (CT) was notable for diffuse uterine enlargement and small-volume ascites.

Further evaluation revealed positive blood and vaginal cultures for GAS. The patient's condition rapidly deteriorated, progressing to TSS, necessitating intubation and vasopressor support. She was promptly initiated on clindamycin and ceftriaxone along with intravenous immunoglobulin (IVIG) and stress-dose steroids. Due to the severity of her condition, the patient was transferred to a tertiary obstetric center for a higher level of care.

**Figure 1:**
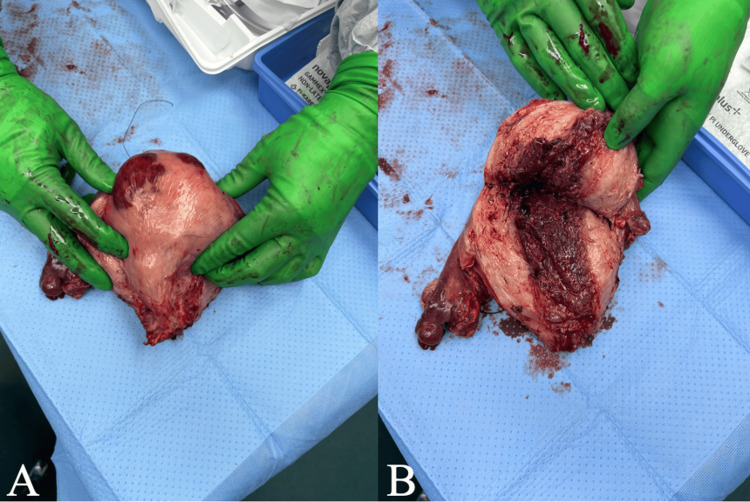
Gross image of the gravid uterus status post-vaginal delivery on day 5 in a 42-year-old female with a peripartum GAS infection (A) Gross uterus exhibiting tan to focally hemorrhagic, smooth serosa with attached bilateral adnexa and a detached cervix. (B) Cut section of the uterus revealing a triangular endometrial cavity with a soft, nodular, brown lining. Acute inflammation involving the cervix and corpus, bilateral acute oophoritis and salpingitis with abscess formation, and diffuse thrombosis involving vessels in the uterus and bilateral adnexa are evident. GAS, group A streptococcus

Within hours of arrival, the patient underwent an urgent exploratory laparotomy, total abdominal hysterectomy with bilateral salpingo-oophorectomy (TAH/BSO), and abdominopelvic washout. Intraoperative findings included an enlarged uterus consisting of a gravid uterus (Figure 1). The patient was also noted to have mucopurulent peritoneal fluid. She had a large pale and necrotic uterus approximately 20 weeks in size with noted developing areas of Couvelaire uterus. Her bilateral ovaries and omentum were coated with green mucopurulent discharge.

A repeat TTE raised concern for stress-induced cardiomyopathy. On post-operative day 4 (POD4), focal seizure-like activity was observed. A CT of the head revealed a left subdural fluid collection suggestive of an empyema with a midline shift. Additionally, the patient exhibited signs of hepatic and renal dysfunction and lactic acidosis (Table 1). 

**Table 1:** Laboratory findings with reference ranges on post-operative days 4-6 BUN, blood urea nitrogen; eGFR (CKD-EPI), estimated glomerular filtration rate (using the Chronic Kidney Disease Epidemiology Collaboration equation);  AST, aspartate aminotransferase; ALT, alanine aminotransferase

	Reference range and units	Post-operative Day 4	Post-operative Day 5	Post-operative Day 6
Lactic acid, plasma	0.5-2.0 mmol/L	8.1	4.1	2.4
		5.0	4.8	2.5
		5.3	5.7	4.6
		5.8	7.0	
		5.2		
Glucose 1	70-99 mg/dL	166	120	102
		144	126	104
		219	159	168
		189	167	133
BUN	6-20 mg/dL	30	22	18
		29	26	19
		34	29	20
		36	29	23
Creatinine, serum	0.50-0.90 mg/dL	0.76	0.74	0.68
		0.74	0.81	0.74
		0.82	0.80	0.76
		0.83	0.84	0.88
eGFR (CKD-EPI)	≥60 mL/min/1.73 m^2^	100	>100	>100
		>100	93	>100
		92	94	100
		90	89	84
Sodium	136-145 mmol/L	150	144	145
		150	147	148
		146	148	146
		146	150	145
Potassium	3.5-5.2 mmol/L	4.3	4.5	3.9
		3.6	3.4	4.1
		3.3	3.3	3.4
		3.5	4.0	4.2
Chloride	98-107 mmol/L	106	107	109
		105	105	110
		102	106	109
		104	108	107
CO_2_	22-30 mmol/L	26	27	27
		32	30	28
		32	30	27
		31	25	27
Anion gap	7-16 mmol/L	18	10	9
		13	12	10
		12	12	10
		11	17	11
Calcium	8.4-10.3 mg/dL	7.9	7.3	7.2
		7.1	7.5	7.4
		7.1	8.2	7.4
		7.1	7.6	7.5
total protein	6.4-8.3 g/dL	5.2	4.9	5.4
		5.1	5.9	5.5
		4.9	5.1	
Albumin (g/dL)	3.5-5.2 g/dL	2.0	2.3	2.4
		2.1	2.6	2.6
		2.0	2.4	
Total bilirubin	0.0-1.2 mg/dL	1.4	1.2	1.3
		1.5	1.6	1.1
		1.6	1.1	
Alkaline phosphatase	35-104 U/L	134	122	137
		103	153	135
		91	133	
AST	0-32 U/L	712	177	107
		750	396	147
		726	479	
ALT	0-33 U/L	628	271	294
		677	533	301
		662	538	

**Figure 2:**
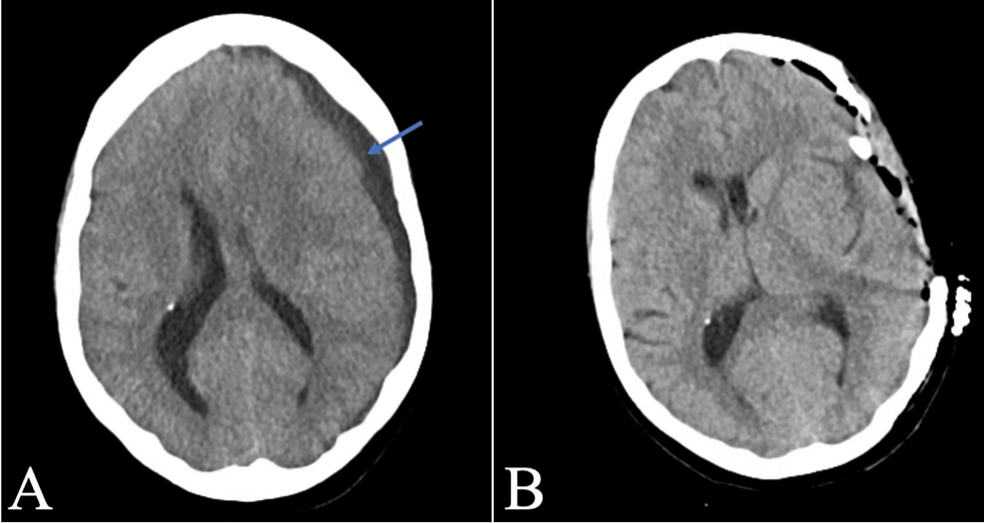
Radiographic images of a non-enhancing subdural empyema in a 42-year-old female with a remote history of seizure disorder and congenital deafness (A) Preoperative axial view of a non-contrast CT of the head showing an 8.8 mm low-density subdural fluid collection identified at the left frontotemporal and parietal levels. There is mass effect with effacement of sulci and effacement of the left lateral ventricle level of the frontal horn. There is a left-to-right midline subfalcine shift of 7 mm. Non-enhancing subdural collection is outlined with a blue arrow. (B)  Axial views of a post-operative non-contrast CT of the head, two hours post-operative, demonstrating a left-sided decompressive craniectomy and interval resolution of the previous subdural collection, with left-sided extra-axial drainage catheter in place, expected post-operative pneumocephalus, and trace extra-axial blood products. CT, computed tomography

The patient was promptly transferred to the neurosurgical intensive care unit for neurosurgical intervention. A left frontotemporoparietal decompressive hemicraniectomy and evacuation of the SDE were performed on POD4. Due to the development of superimposed cardiogenic shock, the patient was transferred to the cardiac ICU for mechanical circulatory support (MCS). Follow-up CT of the head showed improvement after decompression (Figure 2). The patient's hemodynamic instability necessitated the placement of an intra-aortic balloon pump (IABP) to provide MCS.

Neurology managed the patient's antiepileptic regimen using continuous EEG (cEEG) surveillance to guide antiepileptic medication adjustment. The patient's condition gradually improved, leading to a reduction in seizure activity. Unfortunately, within the following week, the patient suffered acute hypoxic respiratory failure, necessitating emergent intubation.

Over the next few weeks, she made gradual strides toward recovery. Her cardiac function improved, and she tolerated progressive weaning of MCS with eventual IABP removal. This was followed by a de-escalation of ventilatory support with subsequent successful extubation. She continued to progress clinically and was ultimately discharged to an inpatient rehabilitation facility.

The multidisciplinary care employed played a crucial role in navigating the complex nature of her case. The collaboration among disciplines allowed for a comprehensive approach to her treatment, ultimately facilitating her recovery.

## Discussion

Epidemiology of postpartum group A streptococcus infections

The presented case underscores the critical importance of understanding the epidemiology of postpartum GAS infections in the context of maternal health. Historically, outbreaks of postpartum infections were linked to healthcare workers transmitting GAS [4]. While most pregnancy-associated GAS infections are now community-acquired, the annual incidence of GAS vaginal colonization among asymptomatic patients is one per 3,472 deliveries [14]. Postpartum women have a 20-fold higher risk than their non-pregnant counterparts [11]. This case serves as a stark reminder that pregnant and postpartum women remain at a heightened risk, particularly during the immediate postpartum period. The temporal pattern observed in this patient aligns with existing literature, with up to 85% of cases emerging within the first four days after vaginal delivery [4]. The patient's rapid deterioration and progression to TSS highlight the need for heightened awareness and early diagnosis during this vulnerable period.

Pathophysiological insights into postpartum GAS infections

In the context of the presented case, the pathophysiological insights into postpartum GAS infections take on a direct relevance. The intricate interplay of compromised mucosal barriers, changes in vaginal pH, and suppressed innate immunity contributes to the heightened susceptibility of pregnant and postpartum women to GAS infection [3]. The M protein, a major virulence factor, and GAS toxins play pivotal roles in disease progression. GAS strains with abundant M protein resist phagocytosis, while toxins induce inflammatory cytokine production, leading to hypotension, capillary leakage, and multiorgan failure [3]. The patient's multiorgan involvement and clinical deterioration align with the cytokine-induced capillary leakage and multiorgan failure characteristic of severe GAS infections. Genetic factors and specialized immune responses associated with female reproductive tract tissues also influence disease outcomes [10]. This case thus exemplifies how various patient-specific factors can interact with the pathophysiological mechanisms described in the literature, leading to the complex clinical presentation observed.

Clinical presentation and challenges in diagnosis

The clinical presentation of postpartum GAS infections is variable, encompassing a broad spectrum of symptoms that can mimic various other medical conditions. Initially, subtle manifestations, such as fever, nausea, vomiting, and myalgia, may rapidly evolve into more severe outcomes such as septic shock and necrotizing fasciitis, as illustrated in the literature [11]. The challenges associated with diagnosing postpartum GAS infections become more pronounced when closely examining the patient's clinical presentation. Her initial symptoms, which included abdominal pain and vaginal bleeding, could potentially be misinterpreted as part of the usual postpartum recovery process, thereby obscuring the underlying presence of a severe infection. The swift progression to TSS, accompanied by the subsequent complications of cardiac dysfunction and SDE, further underscores the unpredictable nature of this condition and highlights the imperative need for vigilance. The range of clinical features exhibited in GAS infections can readily mimic other medical conditions, leading to diagnostic delays. This complexity is mirrored in the patient's case, where laboratory abnormalities such as leukopenia and an increased lactic acid concentration provided valuable diagnostic insights [8]. Consistent with existing literature, diagnostic measures such as blood and urine cultures, lactic acid, endometrial aspiration, and CT imaging played a crucial role in identifying the source of infection and determining disease severity [11]. Consequently, recognizing GAS infections as a potential component of the differential diagnosis for postpartum females, particularly in the presence of atypical symptoms, remains essential, as early detection is pivotal for averting mortality.

Multidisciplinary approach to management

The multidisciplinary approach to managing postpartum GAS infections presented in this case resonates deeply with the literature's emphasis on interdisciplinary collaboration. The patient's complex clinical course necessitated the involvement of obstetrics, critical care/ICU, infectious diseases, cardiology, neurosurgery, and neurology, mirroring the collaborative efforts advocated in the literature. This case exemplifies the necessity of tailored treatment strategies that address the unique challenges posed by each patient, given the rare yet severe sequelae of infectious spread demonstrated. The prompt initiation of antibacterial agents, antipyretics, and supportive therapies with expeditious surgical intervention aligns with established treatment recommendations. Although there is controversial evidence for use, IVIG was administered in this patient along with stress-dose steroids and MCS, reflecting the literature's focus on targeted interventions to counteract the cytokine-induced systemic effects and multiorgan involvement of severe GAS infections. The stepwise approach to management, involving both surgical and medical interventions, mirrors the comprehensive strategies discussed in the literature to ensure optimal patient outcomes.

Prognosis and future directions

This patient's presentation serves as a reminder of the multifaceted nature of the prognosis of postpartum GAS infections. The timely interventions and multidisciplinary approach likely contributed to her favorable outcome, underscoring the significance of early recognition and intervention. The case's complexity and potential for rapid deterioration highlight the ongoing need for research to further elucidate the underlying mechanisms and refine treatment strategies. The unique interplay of factors, such as disease onset and infecting GAS strain, are exemplified in this case and emphasize the necessity of individualized prognostication. The resurgence of severe postpartum GAS infections, despite medical advancements, reinforces the importance of ongoing research to uncover evolving contributing factors and vulnerabilities within this vulnerable population. Thus, this patient's journey not only sheds light on her specific case but also contributes to the broader understanding of the prognosis and future directions in the management of postpartum GAS infections.

## Conclusions

In conclusion, this case serves as a poignant reminder of the complexities inherent in the management of postpartum GAS infections. This clinical course highlights the rapid and severe progression of the disease and underscores the critical importance of interdisciplinary collaboration in providing comprehensive patient care. The lessons derived from this case highlight the importance of individualized patient care, adaptive treatment strategies, and ongoing research to enhance our understanding of this rare yet potentially devastating obstetric complication. As the medical community continues to strive for improved maternal outcomes and reduced maternal morbidity and mortality, it is paramount to remain vigilant in recognizing the signs and symptoms of postpartum GAS infections. Further investigations are warranted to delve deeper into this condition's pathophysiology, risk factors, and therapeutic approaches.
